# Framework Flexibility Governs Molecular Accessibility and Selectivity in Zeolite Catalysts

**DOI:** 10.1021/jacsau.6c00067

**Published:** 2026-03-18

**Authors:** Luiza M. Manente, Gabriel B. Báfero, Angie L. P. Morales, Carlos O. Ramirez, Jiangtao Zhao, Marlon M. Silva, Amélie Rochet, Florian Meneau

**Affiliations:** a Brazilian Synchrotron Light Laboratory (LNLS), Brazilian Center for Research in Energy and Materials (CNPEM), Campinas 13083-100, Brazil; b Institute of Chemistry (IQ), University of Campinas (UNICAMP), Campinas 13083-970, Brazil; c Brazilian Nanotechnology National Laboratory (LNNano), Brazilian Center for Research in Energy and Materials (CNPEM), Campinas 13083-100, Brazil; d The European Synchrotron Radiation Facility, 71 Avenue des Martyrs, Grenoble 38000, France

**Keywords:** framework flexibility, zeolite catalysis, reaction confinement, ethanol dehydration, operando characterization, Bragg coherent diffraction imaging

## Abstract

Crystalline microporous zeolites are pivotal materials in the petrochemical industry, catalysis, separation, and energy conversion. Their performance arises from ordered frameworks that govern molecular transport and reactivity, complemented by tunable acidity. Although traditionally regarded as rigid, zeolite frameworks display remarkable structural flexibility under working conditions, a feature often overlooked but crucial for understanding their catalytic activity. Here, we report the direct imaging of nanozeolite flexibility during ethanol conversion using *in situ* Bragg coherent diffractive imaging (BCDI). We directly visualize and quantitatively measure zeolite flexibility through its interconnected manifestations as lattice distortion and strain. Three-dimensional strain maps reveal dynamic, facet-specific distortions that correlate with the anisotropic channel orientations of the MFI framework, establishing a link among directional flexibility, molecular selectivity, and catalytic performance. Our measurements achieve picometer-level strain sensitivity and picojoule-scale deformation energy quantification, providing unprecedented insight into the energetics of zeolite framework elasticity. These findings challenge the rigid-host paradigm and establish flexibility as a major parameter for tuning molecular accessibility and selectivity. More broadly, they demonstrate how coherent X-ray imaging can capture real-time lattice dynamics in complex materials, paving the way for adaptive catalysts and advanced separation processes that harness the structural flexibility for enhanced performance.

## Graphical abstract

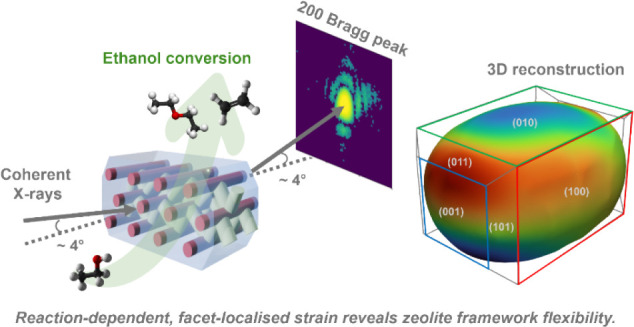

## Introduction

Zeolites are crystalline microporous materials central to catalysis due to their thermal stability, tunable Bro̷nsted acidity, and shape-selective frameworks.^1,2^ Composed of corner-sharing TO_4_ tetrahedra (T = Si, Al), their ordered channels and cavities confine reactants and transition states with subnanometer precision.^3^ Zeolite rigid frameworks are in fact known to exhibit reversible distortions under stimuli such as adsorption, temperature, or pressure.^4,5^ This structural flexibility, expressed as local lattice strain, can modulate adsorption, diffusion, and catalytic turnover.^6^ In nanosized zeolites,^7^ where external surface area and accessible active sites are enhanced, such strain effects are expected to play an important and still underexplored role in catalytic response.

Among zeolitic materials, ZSM-5 (MFI topology) stands out for its versatile catalytic behavior and structural robustness.^8^ Its framework, built from intersecting straight and sinusoidal 10-membered ring channels, creates an anisotropic pore system that governs both diffusion and selectivity.^8,9^ The latter makes ZSM-5 an ideal model to study how directional distortions within its lattice, arising from framework flexibility, can locally modulate diffusion and reaction pathways. In addition, subtle variations in framework composition, such as aluminum or silicon zoning, have shown to alter local polarity and acid-site density, creating nanoscale domains that influence mass transport and catalytic lifetime.^10,11^

A striking manifestation of zeolite flexibility was revealed by the remarkable selectivity enhancement achieved in flexible frameworks. For instance, pure-silica ITQ-55 zeolite^12^ exhibits over 2 orders of magnitude higher selectivity for ethylene over ethane due to a reversible expansion of the minimal pore aperture from 240 to 310 pm. This dynamic pore adjustment allows admission of molecules nearly one ångström larger than the nominal crystallographic opening, underscoring the impact of framework adaptability on molecular discrimination. Comparable phenomena are found in other frameworks with adaptable structures, such as ferrierite (FER)^13,14^ and chabazite (CHA),^15,16^ in which small cooperative distortions of the TO_4_ network adjust pore apertures in response to adsorbates or temperature fluctuations. Similar principles apply to ZSM-5, where subångström anisotropic distortions can modulate channel apertures and diffusion barriers along the straight and sinusoidal directions.^10,17^ Variations in framework composition alter diffusion free-energy profiles by several kBT units, reshaping molecular trajectories through the pore intersections and influencing product selectivity.^10^

Lattice strain, defined as the atomic-scale deviation of a crystal from its ideal structure, is ubiquitous in nanomaterials and critically influences their functional behavior.^18–20^ Despite extensive structural investigations by diffraction^21^ and spectroscopy,^22–24^ measurements of local strain in zeolites have remained largely indirect and ensemble-averaged, lacking the spatial resolution to distinguish bulk from surface distortions or to follow real-time dynamics under reactive conditions. Computational studies have provided valuable insight,^25^ yet experimental access to spatially resolved strain evolution in working catalysts remains a major challenge.

Bragg coherent diffractive imaging (BCDI) overcomes these limitations by providing nondestructive three-dimensional mapping of lattice displacement and strain with nanometer spatial resolution and picometer strain sensitivity.^26,27^ By reconstructing real-space phase maps from coherent diffraction patterns, BCDI reveals facet-specific distortions and their evolution within single nanocrystals under operando conditions.^19,28^ Such measurements have been used to capture zeolite Y framework flexibility^29^ and strain development of adsorption of hydrocarbons in the Cu-ZSM-5 crystal^30^ and to investigate the internal deformations originating from the inhomogeneous Cu ion distributions in Cu-exchanged ZSM-5 zeolite crystals during the deoxygenation of nitrogen oxides with propene.^31^

Here, we employ in situ BCDI to visualize, for the first time, three-dimensional strain dynamics in an individual H-ZSM-5 nanozeolite crystal during ethanol dehydration, a prototypical acid-catalyzed reaction relevant to biomass upgrading. Ethanol acts as both a reactant and structural probe, enabling direct observation of reaction-induced distortions. The resulting facet-dependent and heterogeneous strain fields demonstrate that directional flexibility is a governing factor in molecular diffusion and selectivity, thereby linking nanoscale flexibility to catalytic performance.

## Results and Discussion

### H-ZSM-5 Structure and Catalytic Performance

H-ZSM-5 nanocrystals (Si/Al = 33) were employed in this study. This zeolite features intersecting straight and sinusoidal channels that provide shape-selective access to reactants and products: the straight channels are oriented along the *y*-axis, while the sinusoidal channels lie in the *x*–*z* plane, as illustrated in Figure 1. The nanocrystals were synthesized following Mochizuki et al.^32^ using a concentrated gel to limit crystal growth (see the Supporting Information, Methods), yielding uniform particles (392 ± 40 nm), with high crystallinity. X-ray diffraction (XRD) analysis confirms the expected MFI framework topology, as shown in Figure S1, and the particle morphology imaged with scanning electron microscopy (SEM) is presented in Figure S2. The catalytic performance was evaluated in the gas-phase ethanol upgrading reaction at atmospheric pressure.

**Figure 1:**
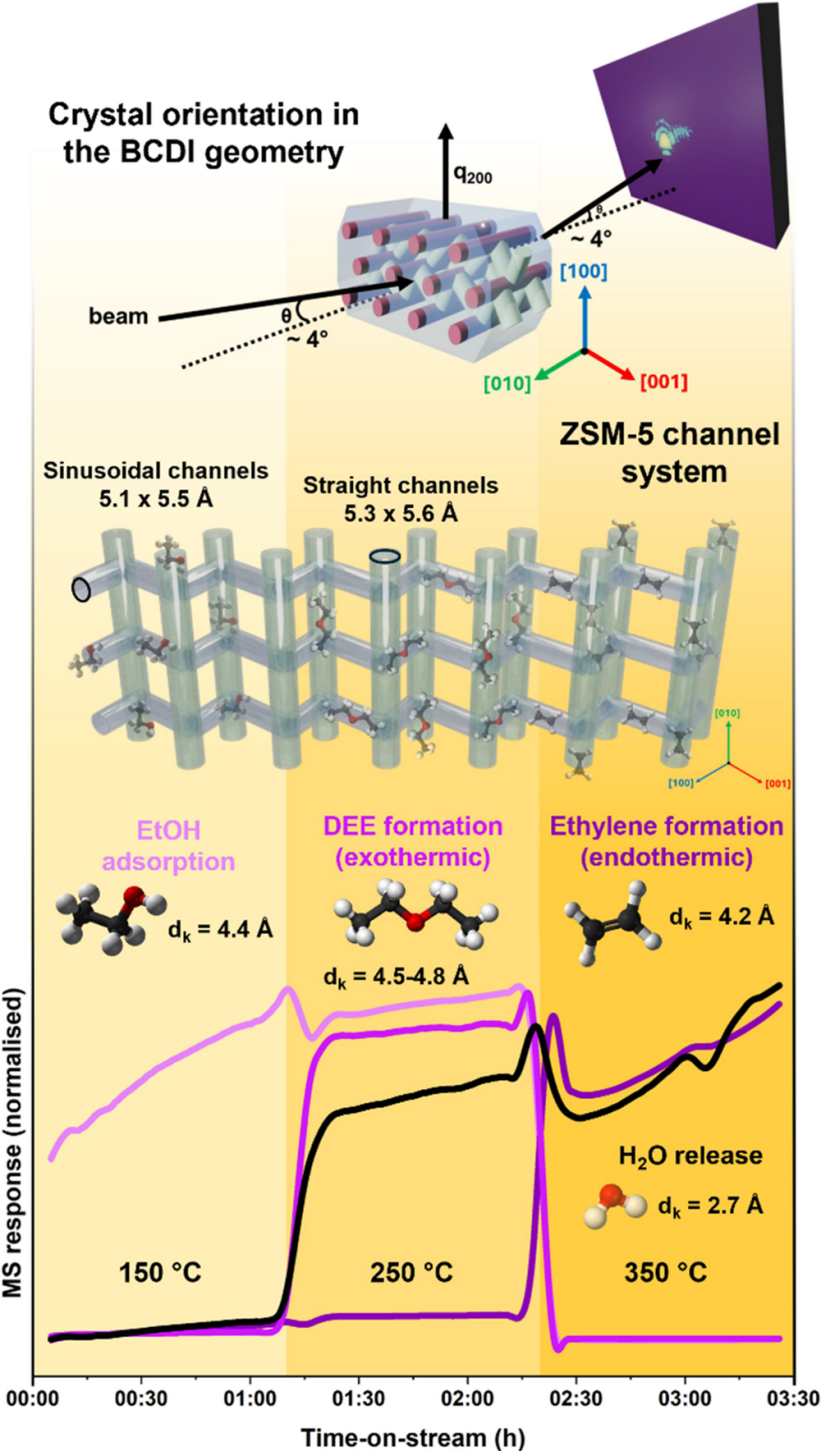
Schematic overview linking the crystallographic orientation of the ZSM-5 nanocrystal used in the *in situ* BCDI experiment, the interconnected straight and sinusoidal channels of the MFI framework, and the sequence of elementary steps in the ethanol dehydration reaction. The structural model illustrates the alignment of the crystal relative to the probed Bragg reflection. The channel architecture is shown with representative molecular configurations, indicating the kinetic diameters (*d*_k_) of ethanol (4.4 Å), diethyl ether (4.5–4.8 Å), ethylene (4.2 Å), and water (2.7 Å) as they diffuse and react within the 5.1 × 5.5 Å sinusoidal and 5.3 × 5.6 Å straight channels. The lower panel displays the corresponding time-on-stream mass spectrometry signals for ethanol (EtOH), diethyl ether (DEE), ethylene, and water (H_2_O) overlaid onto the temperature-dependent regime map (light gray for He, light-to-dark yellow gradients for increasing EtOH-reactive conditions). Ethanol adsorption dominates at 150 °C, followed by exothermic diethyl ether formation at 250 °C and endothermic ethylene formation at 350 °C.

Vaporized ethanol was delivered using He as a carrier gas, and the reaction effluent was analyzed online by mass spectrometry (MS) and gas chromatography (GC) (see the Supporting Information, Meethods). The dehydration of ethanol on acid catalysts proceeds via two parallel routes: (i) bimolecular condensation yielding diethyl ether and water, dominant at moderate temperatures, being exothermic (−25.1 kJ mol^–1^)^33^ and (ii) monomolecular elimination forming ethylene and water, which prevails at higher temperatures, being endothermic (+44.9 kJ mol^–1^).^33^ Both pathways are catalyzed by Bro̷nsted acid sites (BAS). The MS response is presented in Figure 1. At 150 °C, ethanol remained largely unconverted, while at 250 °C, approximately 50% conversion was achieved, producing mainly diethyl ether and water. Increasing the temperature to 350 °C led to near-complete conversion to ethylene and water, consistent with the dual Bro̷nsted acid-catalyzed dehydration pathway observed for H-ZSM-5.^33^ These macroscopic trends are well established: at low temperatures, the reaction is diffusion-limited and stabilized by hydrogen-bonded surface ethanol and ethoxy intermediates;^34^ at higher temperatures, intraporous diffusion accelerates and the monomolecular route dominates.^34^ However, the connection between the reaction progression and local lattice strain remains largely inaccessible to ensemble methods. To address this, *in situ* BCDI was employed to directly visualize the evolution of lattice distortions and nanoscale strain in individual H-ZSM-5 nanocrystals under reactive environments, thereby linking catalytic performances to the dynamic internal structure of the zeolite.

### Evolution of the Nanozeolite Lattice and Strain under a Reactive Environment

Bragg coherent diffractive imaging (BCDI) was used to resolve the morphology and projected three-dimensional (3D) displacement field within an individual nanozeolite crystal under the reaction conditions. *In situ* measurements were performed using the reactor developed by Richard et al.^35^ The BCDI experiment is schematically shown in Figure 1. The coherent diffraction patterns, presented in Figure 2, provide direct insights into how the lattice of an individual H-ZSM-5 nanocrystal responds to external stimuli, i.e., temperature, gas adsorption, and reactive atmospheres. The first diffraction row, corresponding to the central peak projection near the (200) Bragg maximum, captures the distortions and strain along the (200) direction, while the orthogonal 90° reciprocal-space cross sections (2nd and third rows) provide information about the lattice responses along the other directions. Together, these views offer qualitative insight into both isotropic and directional aspects of the lattice response and how strain evolves with increasing temperature and ethanol exposure.

**Figure 2:**
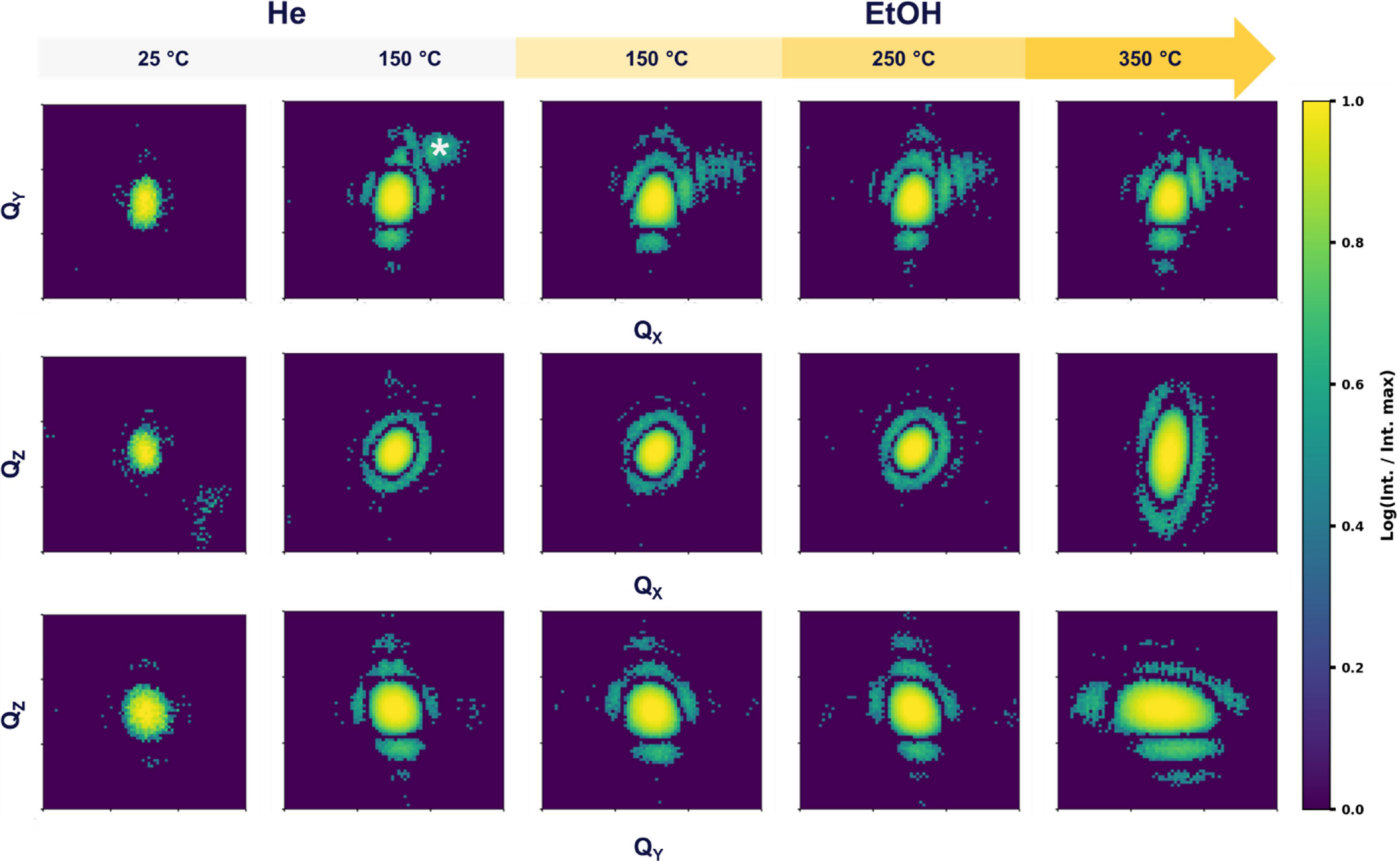
Coherent X-ray diffraction patterns from a single H-ZSM-5 nanocrystal collected under inert He (light gray panels) and reactive ethanol atmospheres (light-to-dark yellow gradient, increasing with temperature). The top row shows the central Bragg peak, while the second and third rows present orthogonal 90° cuts through reciprocal space. Under He, the diffraction patterns remain isotropic, consistent with a coherent and unstrained lattice. Upon ethanol exposure, the patterns broaden, tilt, and elongate anisotropically, indicating facet-dependent distortions driven by adsorbate interaction and reaction. At higher temperatures, the intensity distributions develop pronounced asymmetry, indicative of dynamic strain gradients and heterogeneous lattice response. The asterisk (*) indicates an “alien” signal from a neighboring crystal, which was removed during the reconstruction phase-retrieval step.

Under He, at room temperature (RT) and 150 °C, the diffraction patterns display a symmetric central peak surrounded by well-defined fringes, characteristic of an unstrained crystal. However, a distinct transformation occurs upon the introduction of ethanol. At 150 °C under ethanol, the Bragg peak becomes asymmetric and displays a triangular shape, and additional satellite speckles appear, signatures of adsorbate-induced lattice distortions. These changes indicate that the interaction of ethanol molecules with Bro̷nsted acid sites locally modifies the bonding environment, inducing lattice distortion and strain. This triangular pattern was previously reported^30,31,36^ for ZSM-5 crystals and attributed to residual tetrapropylammonium template molecules remaining from the synthesis.^36^ These chemical residues located in the core of the nanocrystal induce the expansion of the core but contraction of the rim of the zeolite due to different thermal expansion coefficients, leading to the triangular diffraction pattern. However, high temperature calcination procedures,^36^ applied in this work, guarantee complete removal of template residues: the triangular pattern is here solely attributed to ethanol adsorption and catalytic reaction. Moreover, the asymmetry of the diffraction signal reveals that these distortions are strongly directional, preferentially aligned along a specific crystallographic axis associated with external facets or channel orientations.

As the temperature increases to 250 °C, where ethanol conversion produces ∼50% diethyl ether and water, no major evolution of the diffraction patterns is observed. However, at 350 °C with near complete ethanol conversion to ethylene and water, the orthogonal cuts reveal pronounced anisotropy, where the central peaks elongate. These features are attributed to the formation of radial strain gradients and anisotropic relaxation between core and surface regions, possibly indicating a core–shell strain structure. These systematic diffraction changes delineate a full cycle of zeolite response, from a strain-free framework under inert conditions to an adsorbate-distorted and dynamically reorganizing lattice under reaction.

### Three-Dimensional Reconstruction of Nanozeolite Lattice Displacement and Strain Fields

To obtain the 3D amplitude and phase distribution of the nanozeolite, as well as the strain facet anisotropies, iterative phase retrieval algorithms were applied to the BCDI data.^26,27^ The amplitude of the reconstructed image represents the electron density of the sample under study, and the phase corresponds to the projection of the displacement of the crystal lattice along the scattering vector q_200_. Figure 3 shows a 3D reconstruction of the zeolite nanocrystal. The reconstruction provides the 3D shape of the zeolite nanocrystal as well as its lattice displacement and strain with a voxel size of 34 nm^3^. The 3D reconstruction shape and size (403 × 329 × 234 nm^3^) do match with the characteristic shapes imaged with SEM (Figure S2). Moreover, selected area electron diffraction (SAED) analysis (Figure S3) was employed to determine the crystallographic orientation of the nanocrystal. The retrieved electron density map is drawn at an isosurface of 50% of the maximum amplitude. The [100], [010], and [001] crystallographic directions are indicated by blue, green, and red arrows, respectively, and the corresponding crystallographic facets follow the same color scheme. Details of the coherent X-ray diffraction data processing and the procedure used for crystallographic facet determination are provided in the Supporting Information, Methods section.

**Figure 3:**
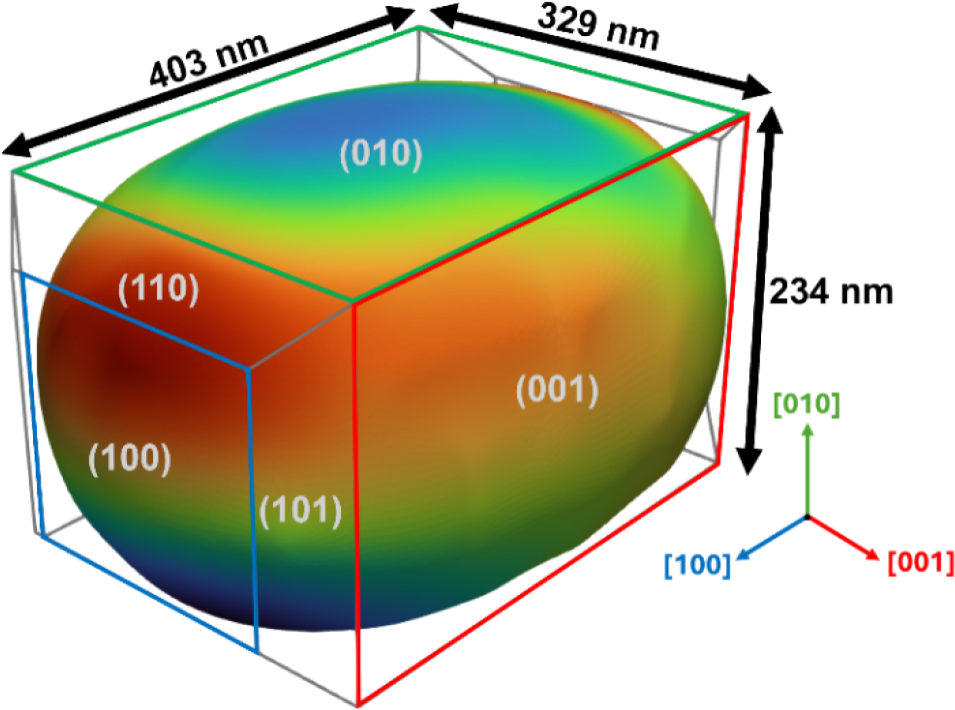
BCDI reconstruction of the H-ZSM-5 nanocrystal showing its morphology, crystallographic facet orientations, and overall dimensions (403 × 329 × 234 nm^3^). The isosurface corresponds to 50% of the maximum electron density, and the surface color map represents the lattice displacement field measured at 150 °C under ethanol. The dominant facets, (010), (100), (001), and their symmetry-related counterparts are shown, and the color-coded axis denotes the [100], [010], and [001] crystallographic directions.

BCDI provides a unique opportunity to visualize *in situ* the lattice and strain dynamics of nanomaterials under operating conditions.^19,29,37–39^ Figure 4a and b presents the cross-sectional maps of lattice displacement and heterogeneous strain within the H-ZSM-5 nanocrystal as a function of temperature and gas environment, revealing the dynamic and anisotropic structural response of the nanozeolite framework to ethanol adsorption and catalytic turnover. The heterogeneous strain captures the spatially varying lattice distortions within the crystal, derived from local phase gradients in the reconstructed image and manifested as broadening or asymmetry of the Bragg diffraction peak. On the other hand, the homogeneous strain refers to the uniform lattice deformation across the crystal, corresponding to an overall change in lattice spacing and observed as a shift in the Bragg peak position. Together, they describe the local and global components of the crystal’s elastic response, as further illustrated in Figure 5.

**Figure 4:**
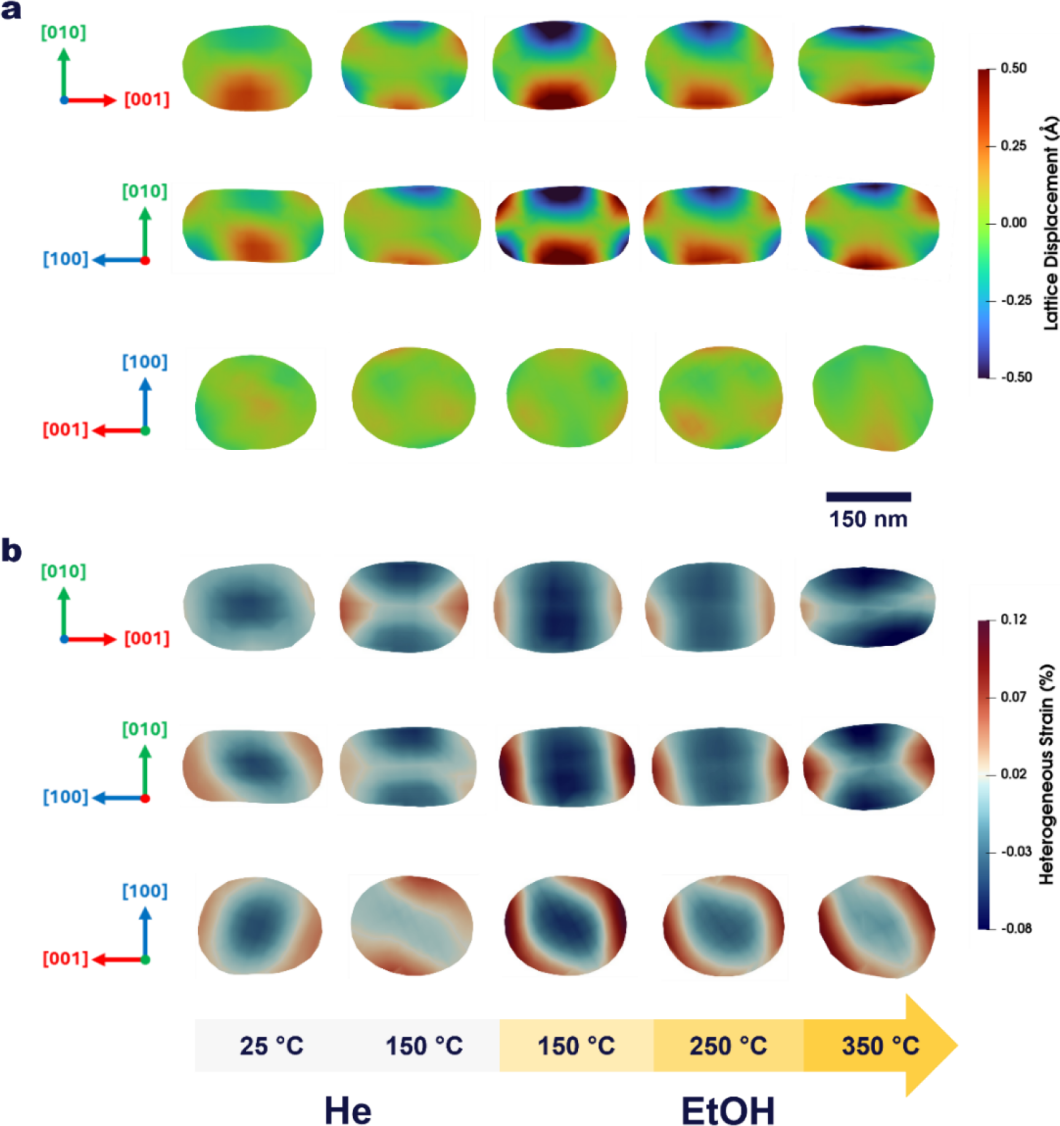
Real-space cross sections of a single H-ZSM-5 nanocrystal showing temperature-dependent (a) lattice displacement (in Å) and (b) heterogeneous strain (in %) across the principal crystallographic orientations ([100], [010], [001]). Under He, both displacement and strain fields remain compact and minimal, characteristic of a relaxed framework. Upon ethanol exposure, localized regions of expansion and contraction emerge in (a), producing pronounced anisotropic displacement patterns that manifest in (b) as facet-dependent tensile and compressive strain domains. These distortions intensify with temperature, giving rise to spatially heterogeneous strain gradients and lattice deformation. Panels follow the same color scheme used throughout the manuscript: light gray for He and light-to-dark yellow gradients for ethanol with increasing temperature.

**Figure 5:**
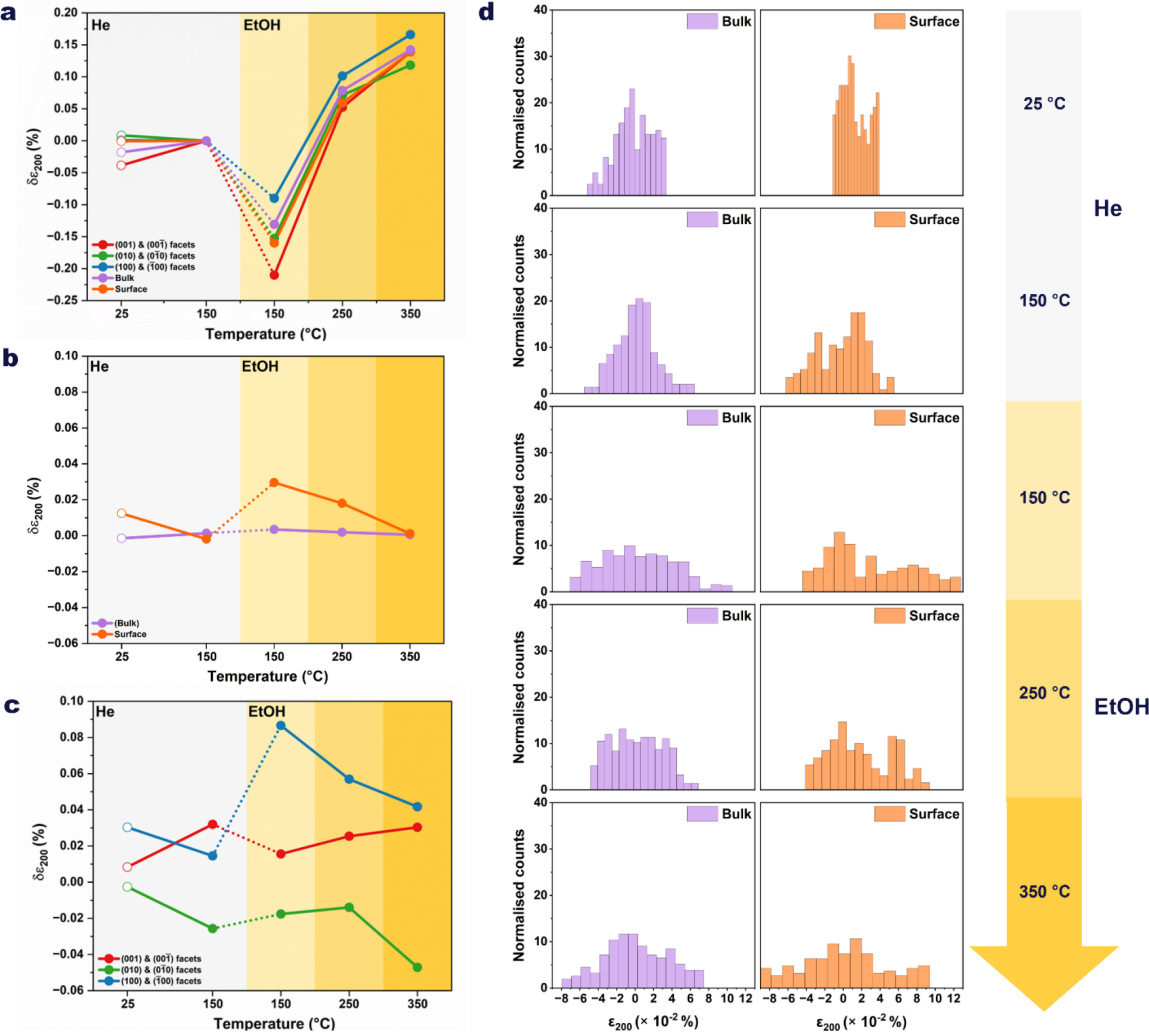
Temperature-dependent evolution of lattice strain in a single H-ZSM-5 nanocrystal under inert and reactive environments. (a) Homogeneous strain (δ_200,homo_) showing the spatially averaged lattice response of the entire crystal as a function of temperature and gas environment. (b) Bulk-surface heterogeneous strain (δ_200,het_), revealing distinct elastic responses during sequential ethanol adsorption and dehydration reaction. (c) Facet-resolved heterogeneous strain for the (100), (010), and (001) facets, highlighting pronounced anisotropies and facet-dependent deformation as reactive conditions intensify. (d) Histograms of heterogeneous strain for bulk and surface regions at each temperature, illustrating the transition from narrow, symmetric distributions under He to broadened and asymmetric profiles under ethanol, consistent with the activation of framework flexibility and localized strain gradients. Panels follow the color scheme used throughout the manuscript: light gray marks measurements under He, while light-to-dark yellow gradients denote increasing temperatures under ethanol.

Figure 5a shows the evolution of the homogeneous strain of the nanozeolite. The strain at 150 °C under He was subtracted to emphasize the relative changes with temperature and gas environments. The bulk, surface, and facet homogeneous strain exhibit similar trends: they become strongly compressive during ethanol adsorption and tensile, when products of interest are formed. This global trend reflects the combined effects of pore filling, adsorbate reorganization, and channel evacuation as the reaction proceeds from ethanol adsorption to diethyl ether formation and ultimately to ethylene production. The heterogeneous strain histograms, presented in Figure 5d, quantify the response of the bulk and surface regions to thermal and chemical perturbations. Under He, both regions show narrow, zero-centered strain distributions, indicating structural equilibrium. Heating to 150 °C under He induces mild strain localized at the [100] and [001] facets (Figure 4b). A continuous strain field connects these facets, suggesting that thermal activation generates a slight tensile stress along the sinusoidal pore network. The bulk strain histogram narrows due to water removal and framework relaxation, while the surface strain broadens, reflecting increased heterogeneities (Figure 5b, d).

Upon ethanol adsorption at 150 °C, the bulk strain becomes strongly compressive, and its distribution broadens, evidencing confinement-driven stress within the pores. Simultaneously, the surface develops tensile strain with a bimodal pattern, arising from opposite facets experiencing tension and compression (Figure 5). This reveals a redistribution of strain between the interior and exterior of the zeolite driven solely by ethanol adsorption. This is accompanied by a sharp increase in both lattice displacement and heterogeneous strain amplitudes (Figure 4a, b), specifically at the [100] and [001] facets. Pronounced lattice distortions also emerge along the [010] direction parallel to the straight channels. This anisotropic deformation indicates that ethanol adsorption preferentially perturbs the straight-channel walls, consistent with molecular confinement and localized adsorption-induced stress. Moreover, the cross-section in Figure 4a along the [001] direction shows a twisted deformation of the crystal, likely originating from ethanol interactions with Bro̷nsted acid sites.

At 250 °C, where ethanol converts mainly to diethyl ether, the bulk strain decreases (Figure 5b), reflecting partial stress release as diethyl ether molecules better fit the pore geometry. In contrast, the surface strain increases and retains a bimodal profile dominated by compressive components associated with surface restructuring, here referring to a reorganization of the near-surface strain field. Figure 4b shows that the heterogeneous strain remains preferentially localized at the [100] and [001] facets.

At 350 °C, under conditions of near-complete ethanol conversion to ethylene, the bulk strain further decreases due to pore evacuation and reduced confinement, while the surface strain reaches its maximum, consistent with high catalytic turnover near the crystal rim. The continuous strain field connecting the [100] and [001] facets indicates that the sinusoidal pore network remains the most strongly affected. This highly anisotropic, facet-selective strain, observed here for the first time, is consistent with enhanced catalytic activity, increased framework flexibility, and a higher density of Bro̷nsted acid sites near the nanocrystal rim, effectively forming a core–shell-like strain pattern. High-angle annular dark-field scanning transmission electron microscopy/energy dispersive X-ray spectroscopy (HAADF-STEM/EDX) mapping (Figure S4) corroborates this interpretation by revealing an inhomogeneous aluminum distribution, with higher aluminum concentration.

Beyond establishing a higher density of Bro̷nsted acid sites at the rim, the observed lattice distortions may also perturb their local geometric and electronic environment. In MFI frameworks, the catalytic proton resides in bridging Al–O–Si groups whose bond angles and local electrostatics are sensitive to subtle framework deformations. Strain induced variations in channel dimensions and Si–O–Al geometry can therefore influence proton accessibility and transition-state stabilization, effectively modulating reaction energy barriers. Recent single-crystal studies on ZSM-5 have emphasized the strong coupling between structural anisotropy, diffusion pathways, and catalytic kinetics.^40,41^

### Elastic Energy Landscape

To quantify the structural changes driven by ethanol adsorption and dehydration, we determined the elastic (deformation) energy landscape using the three-dimensional strain distribution defined by the following equation:

$$E_{s_{200}}=\frac{3}{2}K\int {\left(\frac{\delta u_{200}}{\delta x_{200}}\right)}^{2}dV$$

where *K* is the bulk modulus of H-ZSM-5 (*K* = 26.7 ± 2.0 GPa, see the Supporting Information, Methods), *u*_200_ is the displacement value along the [200], and *x*_200_ is the (200) lattice constant of H-ZSM-5. This formulation captures the strain or deformation energy associated with atomic displacements from equilibrium positions regardless of their origin. Figure 6 shows the resulting elastic energy across the temperature and reaction environments.

**Figure 6:**
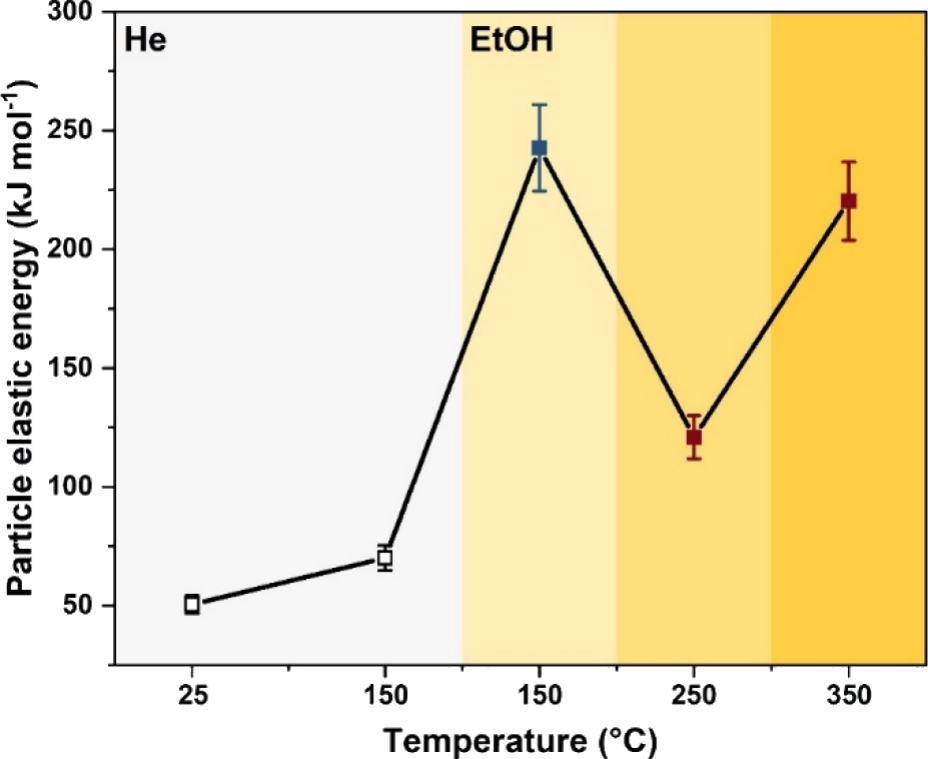
Temperature-dependent particle elastic energy derived from BCDI during sequential exposure to He (light gray) and ethanol (light-to-dark yellow gradients) atmospheres. At 150 °C, the blue square denotes a regime dominated by ethanol adsorption, where compressive strain develops and drives a sharp increase in elastic energy. At 250 °C, the red square reflects a transition to tensile strain as exothermic DEE formation promotes partial lattice relaxation. At 350 °C, tensile strain intensifies again (red square), coinciding with endothermic ethylene formation and framework distortion.

Ethanol adsorption produces significant framework deformation, yielding energies of ∼243 kJ mol^–1^, substantially higher than the baseline values measured under He. While calorimetric and theoretical studies report adsorption enthalpies of ∼85–95 kJ mol^–1^ at 150 °C,^42^ the much larger strain energy reflects the complex coupling between adsorbate–framework interactions and lattice distortion. Operando IR studies^43^ indicate that molecular ethanol, oxonium ions, and surface ethoxy species coexist at this temperature, with ethoxy gradually converting to ethylene upon heating. These adsorbed and reactive ensembles introduce localized mechanical stresses that manifest as alternating compressive and tensile regions near the nanocrystal periphery. This effect is further enhanced in Al-rich domains, which are more hydrophilic and preferentially stabilize polar molecules,^44^ as well as oxonium/ethoxy intermediates that promote dehydration pathways.^45^ As the temperature increases to 250 °C, the strain energy decreases to ∼121 kJ mol^–1^, coinciding with the formation of diethyl ether. Its production occurs through bimolecular coupling of adsorbed ethanol, releasing heat that facilitates framework relaxation. The transition from monomolecular (ethanol–BAS) to bimolecular reaction pathways distributes mechanical stress more evenly across neighboring channels.^46^ Under these conditions, diethyl ether desorption, favored by its higher volatility, also reduces steric crowding within micropores, further alleviating strain.^47^ At 350 °C, the elastic energy increases again to ∼220 kJ mol^–1^ as unimolecular ethanol decomposition to ethylene becomes dominant. This endothermic pathway reintroduces both energetic and mechanical stresses to the framework. The formation and decay of reactive intermediates contribute to significant local rearrangements, particularly through interactions with framework oxygens and associated C–O bond cleavage,^48^ producing pronounced anisotropic strain fields.

The deformation energies quantified by *in situ* BCDI exhibit a comparable magnitude to those reported for the incorporation of isolated Ir atoms and subnanometer clusters within the 10-membered rings of ZSM-5.^25^ Although the chemical nature of Ir-framework bonding differs fundamentally from the interactions governing molecular adsorption, both scenarios reveal a common physical behavior: strong host–guest coupling giving rise to substantial lattice deformation. Our measurements demonstrate that ethanol, ethoxy intermediates, and reaction products impose localized mechanical stresses on the zeolite framework, revealed as an increase in elastic strain energy.^25^ Direct numerical equivalence between metal incorporation and small-molecule adsorption is unexpected due to differences in local coordination, charge distribution, and framework stoichiometry. The convergence in their mechanical signatures underscores a broader principle, where microporous frameworks may respond to diverse guest species through a universal elastic response.

## Conclusions

Our findings demonstrate that structural flexibility in H-ZSM-5 is both anisotropic and highly responsive to the chemical environment. Facet-localized distortions and reaction-dependent strain evolution reveal that adsorbate–framework interactions actively tune the lattice during catalysis. These nanoscale deformations modulate pore apertures and local diffusion pathways, thereby regulating the accessibility and reactivity of acid sites. The direct correspondence between strain patterns and catalytic regime highlights that framework elasticity is not a passive property but a functional parameter governing zeolite behavior under working conditions.

More broadly, these insights provide a structural basis for linking the nanoscale lattice response to catalyst design. Our results show that elastic adaptability regulates molecular transport and reshapes the internal reaction environment under working conditions. The observed strain-adsorbate coupling suggests that framework flexibility may influence transition-state stabilization and catalytic turnover. Controlling this flexibility, through composition, morphology, or defect engineering, offers a route to mitigate diffusion limitations and control local reaction environments. This perspective shifts the focus from rigid, shape-selective frameworks to adaptive porous solids that accommodate, and can exploit, adsorbate-induced deformation to improve activity, selectivity, and stability.

## Supplementary Material



## Notes

Experimental details and methods: powder X-ray diffractograms, electron microscopy, and spectroscopy results of the ZSM-5 nanozeolite (PDF)
