# Supplementary material for: Framework Flexibility Governs Molecular Accessibility and Selectivity in Zeolite Catalysts
Source: JACS Au. 2026 Mar 18;6(4):2489–97. doi: 10.1021/jacsau.6c00067 (PMC13126194; doi:10.1021/jacsau.6c00067)
Supplement: Supplementary file 1 [file au6c00067_si_001.pdf]

Supporting Information

Framework Flexibility Governs Molecular Accessibility and Selectivity in Zeolite Catalysts

Luiza M. Manente [a,b§], Gabriel B. Báfero [a§], Angie L. P. Morales [a], Carlos O. Ramirez [c], Jiangtao Zhao [d], Marlon M. Silva [a,b], Amélie Rochet [a,b], and Florian Meneau [a,b*]

[a] Brazilian Synchrotron Light Laboratory (LNLS), Brazilian Center for Research in Energy and Materials (CNPEM), 13083-100, Campinas, Brazil

[b] Institute of Chemistry (IQ), University of Campinas (UNICAMP), Campinas, Brazil

[c] Brazilian Nanotechnology National Laboratory (LNNano), Brazilian Center for Research in Energy and Materials (CNPEM), 13083-100, Campinas, Brazil

[d] European Synchrotron Radiation Facility, BP 220, F-38043 Grenoble, France

* Email: florian.meneau@lnls.br

§ L.M.M. and G.B.B. contributed equally to this work.

Methods

Zeolite synthesis. The ZSM-5 sample with controlled crystallite size was synthesised using the following procedure [1]. Tetraethyl orthosilicate (TEOS, 16.20 g) was mixed with water (36.25 g) and tetrapropylammonium hydroxide (TPAOH, 40 wt.% in water, 9.78 g). The resulting solution was stirred at ambient temperature for 24 h. Afterward, a separate solution containing water (13.33 g), $Al(NO_3)_3 \cdot 9H_2O$ (0.59 g), and NaOH (0.31 g) was added to the initial mixture. The prepared gel was then crystallized at 443 K for 24 h. The final gel composition had the molar ratio of $1SiO_2$: $0.01Al_2O_3$: 0.25TPAOH: $0.05Na_2O$: $40H_2O$. The solid product was separated by centrifugation at 15,000 rpm. Na-ZSM-5 was obtained by calcining the as-synthesized material to eliminate the TPA^+ species, with a heating rate of 2 K min^{-1} until 823 K followed by a 4 h plateau. The preparation of the acidic H-ZSM-5 sample followed a straightforward ion-exchange procedure. First, the calcined Na-ZSM-5 was suspended in a 0.1 M NH_4NO_3 aqueous solution and stirred at 333 K for 18 h. The solid phase was then separated by centrifugation at 15,000 rpm. To obtain H-ZSM-5, the dried material was calcined, and the NH_4^+ species were thermally decomposed by heating at a 2 K min^{-1} rate until reaching 823 K, where it was maintained for 4 hours, resulting in the formation of H^+ ions. These synthesis and post-synthesis steps are summarised in Scheme S1.

***In situ* Bragg coherent X-ray diffraction imaging.** *In situ* time-resolved Bragg coherent diffractive imaging (Bragg CDI) experiments were performed at the ID01 beamline of the *European Synchrotron Radiation Facility* (ESRF) in Grenoble, France. H-ZSM-5 zeolite powder was ultrasonically dispersed in ethanol, deposited dropwise onto a silicon wafer, and dried under ambient conditions. A 20 nm carbon layer was then applied to the wafer surface to immobilise the zeolite crystals. Measurements were acquired using a Maxipix pixel detector (516 × 516 $pixels^2$, 55 × 55 μm^2) with an incident X-ray energy of 8.9 keV, focused to a 1 × 1 μm^2 beam. Three-dimensional diffraction data were collected via rocking curve scans from −0.75° to +0.75°, with 0.01° angular steps and 150 frames per scan (1 s exposure per frame). A single H-ZSM-5 nanoparticle was first monitored under He at room temperature. As in the catalytic tests, the sample was then pretreated by drying it at 400 °C for 1 h under a He flow (5 mL min^{-1}). The temperature was then decreased to 150 °C, under He, and another H-ZSM-5 nanocrystal is measured with BCDI. The same nanocrystal is then measured under ethanol upgrading reaction conditions at 150,

250, and 350 °C. During the reaction, ethanol was introduced into the reactor using a saturation flask, with He serving as the carrier gas (5 mL min^{-1}, resulting in an ethanol flow rate of approximately 2.4 mL min^{-1}). The full thermal and reactive sequence used during the *in situ* BCDI experiment is outlined in Scheme S1.

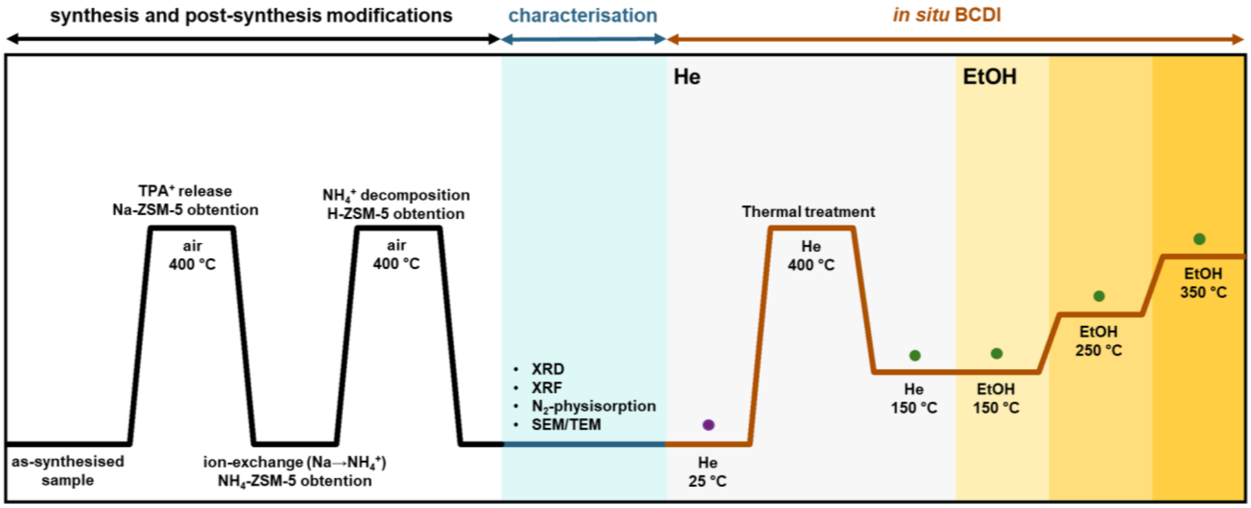

Scheme S1. The schematic illustrates all stages encountered by the material, beginning with the as-synthesised sample and progressing through post-synthesis treatments, characterisation, and the temperature- and atmosphere-dependent sequence used during in situ BCDI. During synthesis and post-synthesis modification (left, white region), the TPA^+ template is removed by calcination at 400 °C in air to obtain Na-ZSM-5, followed by ion exchange ($Na^+ \rightarrow NH_4^+$) and a second calcination step at 400 °C to produce the protonic H-ZSM-5 form. The resulting material is then characterised *ex situ* by XRD, XRF, N_2-physisorption and SEM/TEM (centre, blue region). The right-hand panel summarises the *in situ* BCDI sequence. An initial BCDI dataset is recorded under He at 25 °C, after which the sample undergoes thermal treatment at 400 °C in He. The nanocrystal is then measured at 150 °C under He, followed by measurements at 150 °C, 250 °C and 350 °C under flowing EtOH. The coloured markers denote that different crystals were analysed at specific steps: the purple dot corresponds to the nanocrystal measured at He 25 °C, while the green dots represent the nanocrystals examined at subsequent temperatures under He and EtOH.

Ethanol upgrading reaction. The gas-phase ethanol upgrading reaction was carried out in a plug-flow tubular reactor equipped with a quartz tube. The catalytic bed was composed of 100 mg of H-ZSM-5 placed between two plugs of quartz wool, ensuring stable positioning within the reactor and uniform gas flow. Prior to the reaction, the sample was pretreated by drying at 400 °C for 1 h

under a He flow (50 mL min^{-1}). During the reaction, ethanol was introduced into the reactor using a saturation flask, with He again serving as the carrier gas (50 mL min^{-1}, resulting in an ethanol flow rate of approximately 24 mL min^{-1}). A temperature-programmed reaction was performed to evaluate the catalytic activity at 150, 250, and 350 °C, holding each temperature for 1 h. The gas-phase products were analysed in real time using a triple filter quadrupole HPR-20 Hiden Analytical Mass Spectrometer. Reactants and products were monitored based on mass fragments ranging from 2 to 60 m/z. Fragmentation patterns obtained by electron ionisation were compared with reference data from the National Institute of Standards and Technology (NIST) mass spectrometry database [https://www.nist.gov]. To ensure clarity and avoid overlapping among fragment signals, the following selected mass fragments were used for compound identification: m/z 45 (ethanol), 26 (ethylene), 59 (diethyl ether), 29 (acetaldehyde), 41 (propylene), 18 (water), 4 (helium), and 44 (carbon dioxide). In parallel, reactant conversion and product selectivities were quantified using an online micro-Gas Chromatography (DynamiQ-S, Qmicro), and the corresponding values were calculated according to Eq. S1 and S2, respectively.

$$Conversion = \frac{moles\ of\ consumed\ reactant}{moles\ in\ reactant\ feed} \quad \text{(Equation S1)}$$

$$Selectivity = \frac{moles\ of\ product}{moles\ of\ consumed\ reactant} \quad \text{(Equation S2)}$$

BCDI data reconstruction. Diffraction patterns were reconstructed using a processing pipeline combining PyNX and the cdiutils module [2], implemented in a unified script provided by ESRF. The phase retrieval process employed a sequential iterative approach consisting of 400 RAAR (Relaxed Averaged Alternating Reflections) [3], 300 HIO (Hybrid Input-Output) [4], and 200 ER (Error-Reduction) [5] iterations. To ensure optimal reconstruction quality, 30 independent reconstructions were generated and initialised with random phases. From these, the 10 reconstructions exhibiting the lowest free log-likelihood values [6] were retained. A subsequent filtering step applied the mean-to-max criterion within cdiutils, selecting 5 final reconstructions based on the smallest absolute difference between maximum and averaged electron density. Modal decomposition was then performed on these selected reconstructions. Post-processing procedures included interpolation of the reconstructed object onto an orthogonal grid in the laboratory reference frame, phase unwrapping, and isosurface determination. Phase shift corrections were applied to ensure that the mean phase within the isosurface-bounded region remained zero.

Nanozeolite facets determination. A principal component analysis (PCA) based alignment procedure was applied to the 3D reconstructions of the zeolite crystals. Because the datasets were acquired at different temperatures and times, an axis correction was required to ensure consistent orientation. The principal axis of each reconstruction was determined via PCA using the surface extracted after applying an amplitude threshold, and all datasets were subsequently rotated into a common reference frame. This alignment enabled direct spatial comparison of heterogeneous strain and displacement fields across the various reaction environments. A geometrically consistent wireframe, based on the ideal ZSM-5 framework, was then generated using the PCA-aligned orientation to facilitate crystallographic facet identification. A Python routine was used to assemble and refine the synthetic crystal morphology using the sample's measured dimensions. To correct diagonal artefacts and improve facet planarity, the initial central trapezoidal prism with truncated pyramidal tips along the [100] direction was refined into a shape-adjusted model featuring tunable shrink and truncation parameters, followed by a final correction of edge connectivity. Facet assignment was performed by comparing the orientation and geometry of the reconstructed wireframe to established ZSM-5 crystallographic directions. The elongated axis was indexed as (100), the larger lateral faces as (010), and the oblique truncations as (101), in agreement with the orthorhombic symmetry of the ZSM-5 structure.

Segmentation of the crystal into bulk, surface and facet-specific strain. The data reconstructed from the in situ BCDI datasets had a voxel size of 34 nm. The outermost voxel layer of each reconstruction was defined as the surface, while all interior voxels were classified as the bulk. This segmentation was implemented using a Python script, generating masks for both regions at each temperature step of the catalytic process. Facet-specific strain was obtained by importing the full 3D strain volume into ParaView (http://www.paraview.org) and manually delineating each crystallographic facet using the Select Cells On tool. A fixed-depth selection corresponding to 15 voxels was applied to ensure consistent sampling across all reconstructions, yielding a uniform facet shell from which strain values were extracted. The strain values obtained at the specific voxels for bulk, surface and individual facets were exported for analysis. Statistical treatment and visualisation were performed in OriginPro, using the averaged strain values extracted from each segmented region.

Powder X-ray diffraction, X-ray fluorescence and X-ray photoelectron spectroscopy. Powder X-ray diffraction (XRD) patterns were collected on a Bruker D8 Advance Eco diffractometer (Cu $K\alpha_1$, λ = 1.5406 Å; 40 kV, 25 mA) over 5-55° (2θ) with 0.04° steps at 5° min^{-1}. Elemental composition was assessed semi-quantitatively by micro-X-ray fluorescence (Shimadzu µEDX-1300), analysing five 50 µm spots per sample; quantification ($Z \geq 12$) was performed via the fundamental-parameters method. Elemental composition was also quantitatively determined by X-ray photoelectron spectroscopy (XPS) measurements, that were performed on a Thermo Scientific K-Alpha spectrometer using monochromatic Al Kα radiation (hν = 1486.6 eV). Survey spectra (200 eV pass energy, 1.0 eV step) and high-resolution core-level spectra (50 eV pass energy, 0.1 eV step) were acquired with a 300 µm spot size. Atomic compositions were obtained from background-corrected peak areas using instrument sensitivity factors. The diffractograms (Figure S1) confirm that both Na-ZSM-5 and its proton-exchanged H-ZSM-5 derivative crystallise in the MFI framework, showing the characteristic reflections at 7-9°, 22-25°, and 30-32°. A reproducible ~0.12° shift of all major peaks to higher angles upon ion exchange indicates a slight lattice contraction, consistent with the smaller effective ionic radius of H^+ and the well-known unit-cell shrinkage accompanying conversion of the Na-form. XRF analysis (bulk-sensitive) yields a Si/Al ratio of 33, while XPS analysis yields a Si/Al ratio of 18 (surface-sensitive).

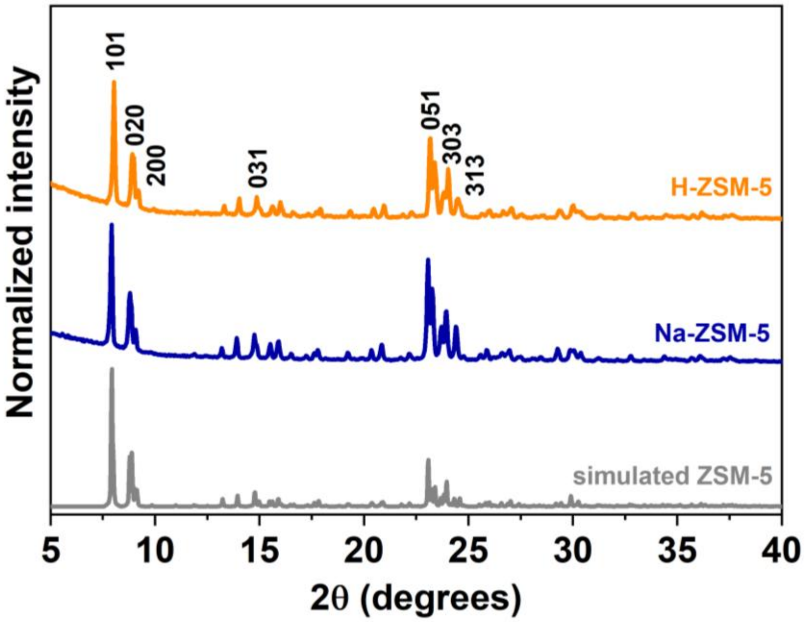

Figure S1. Powder XRD patterns of the as-synthesised Na-ZSM-5 and ion-exchanged H-ZSM-5 nanozeolites, compared with the simulated MFI pattern [https://www.iza-online.org].

Scanning electron microscopy. Scanning electron microscopy (SEM) was performed using a FEI Inspect F50 microscope (LNNano/CNPEM). The ZSM-5 powder was dispersed in ethanol, drop-cast onto a silicon wafer, and coated with ~4 nm of carbon prior to imaging. SEM analysis reveals that the material consists of uniform prismatic nanocrystals with MFI-type facets (Figure S2a). The particles show smooth external surfaces and no signs of secondary nucleation, indicating controlled hydrothermal growth. A statistical evaluation of 100 crystals (ImageJ) yields an average particle size of 392 ± 40 nm (Figure S2b).

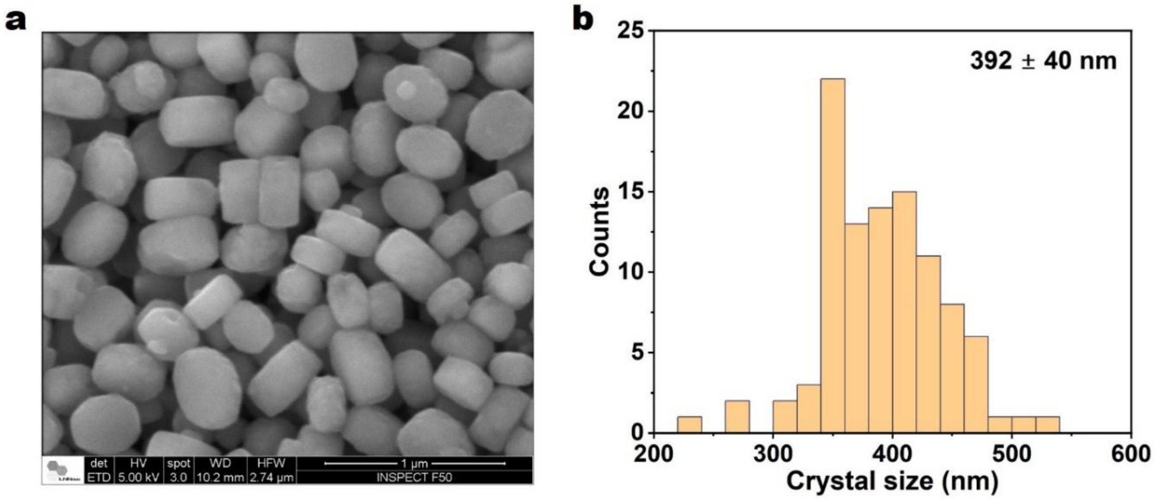

Figure S2. (a) SEM image of the as-synthesised nano-ZSM-5 crystals and (b) corresponding size distribution histogram obtained from ImageJ analysis of 100 particles. The crystals display an average size distribution centred at 392 ± 40 nm.

Selected-Area Electron Diffraction. Four particles were analysed by Selected-Area Electron Diffraction (SAED) (Figure S3) using a 40 μm diameter selected-area aperture positioned in the image plane of the objective lens post-field. The resulting diffraction patterns were indexed close to the [010] zone axis (ZA). Distinct diffraction spots corresponding to specific crystallographic planes were identified (red: (001), green: (101), yellow: (100)), and g-vector magnitudes were measured using the Velox Processing software (FEI Company/Thermo Fisher Scientific). The H-ZSM-5 particles exhibited pronounced beam sensitivity even under cryo-electron microscopy dose conditions (ca. 4 $e^{-}/Å^{2}s$), requiring rapid orientation and immediate acquisition without perfect zone-axis alignment.

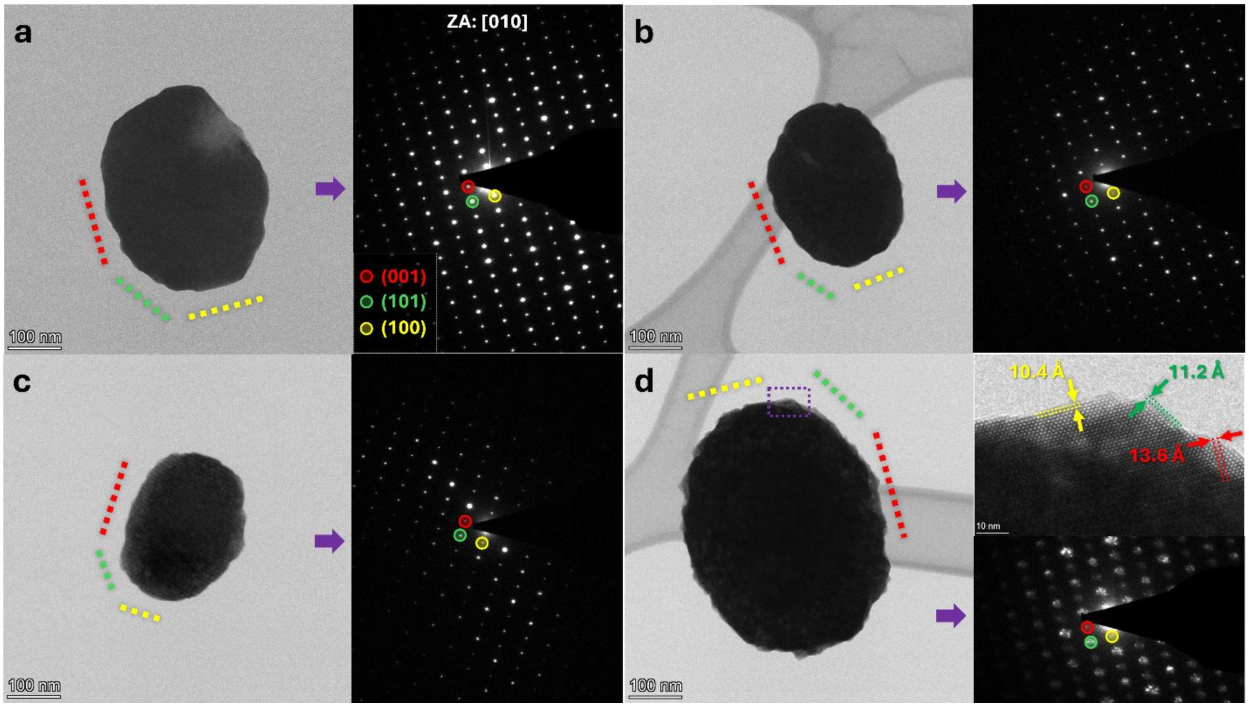

Figure S3. SAED analysis of four different H-ZSM-5 particles (right panels, a–d) and their corresponding diffraction patterns (left panels, a–d), indexed close to the [010] zone axis (ZA). The inset in the upper right corner of panel d shows a magnified view of the pore structure of the region indicated by the dashed purple rectangle.

Transmission electron microscopy. Transmission electron microscopy (TEM) specimens were prepared by drop-casting 0.3 μL of the nanocrystal suspension, diluted in Milli-Q water and ultrasonicated for 5 min, onto lacey carbon grids coated with an ultrathin carbon film. Grids were glow-discharged (Pelco easiGlow) prior to deposition and dried under ambient conditions. Imaging was carried out on a double aberration-corrected FEI Titan Themis microscope (LNNano/CNPEM) equipped with a SuperXG1 EDX detector, operated in STEM mode at 300 kV (17.9 mrad convergence, 115 mm camera length). HAADF-STEM imaging and EDX mapping performed on independent ZSM-5 nanocrystals (Figure S4a-d) reveal reproducible inhomogeneous aluminium distribution. The Si Kα maps display a uniform framework signal across each particle, while the Al Kα maps exhibit clear spatial gradients. Line-scan profiles extracted along indicated directions confirm this phenomenon.

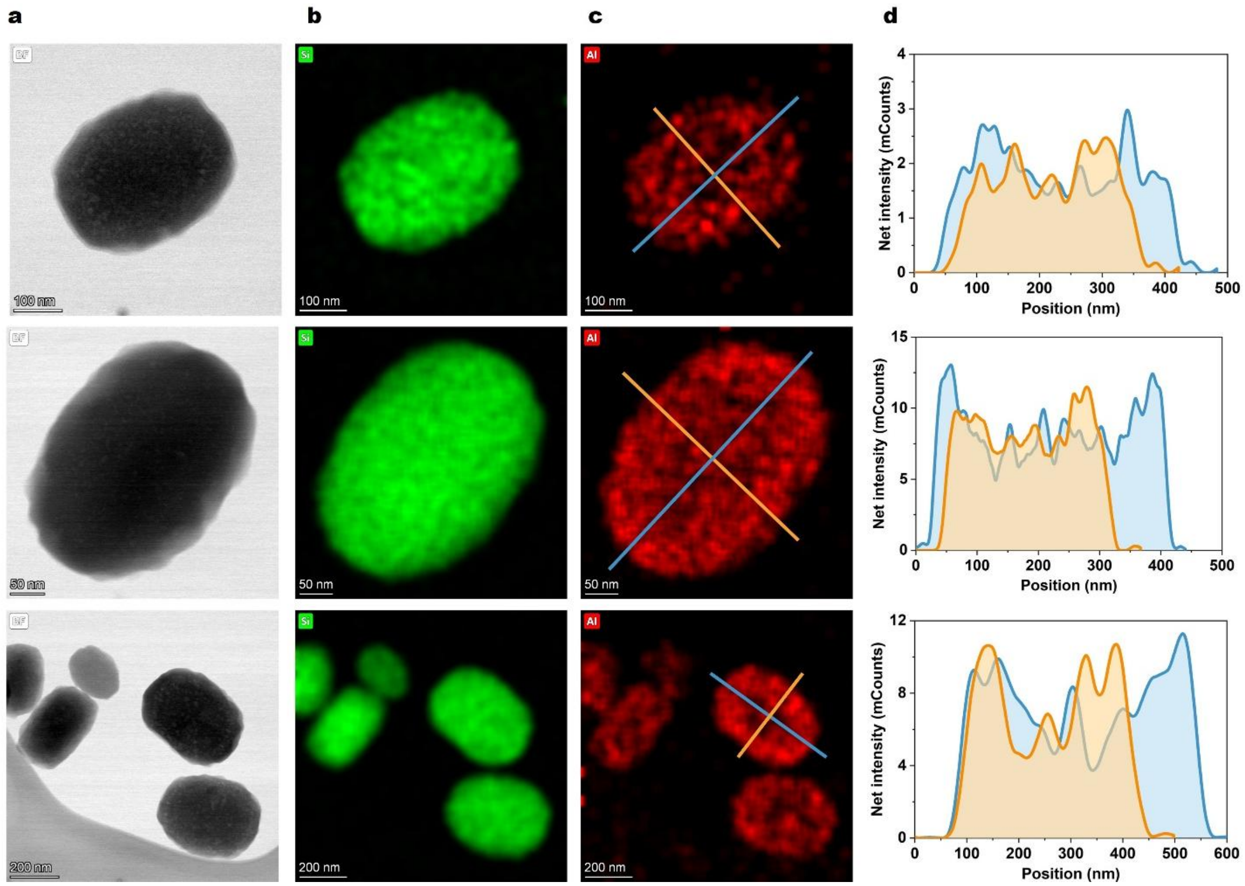

Figure S4. (a) HAADF-STEM images of three representative nano-ZSM-5 crystals. (b) Corresponding Si Kα EDS elemental maps. (c) Al Kα EDS maps of the same crystals, overlaid with the line profiles (blue and orange lines) used to quantify aluminium distribution across the particle. (d) EDS line-scan intensity profiles extracted along the indicated directions, showing the spatial variation in Al concentration relative to Si.

Estimation of the bulk modulus for H-ZSM-5 (Si/Al = 33). No direct measurement of the bulk modulus (K) is available for high-silica H-ZSM-5 under penetrating-medium conditions. We estimated the bulk modulus K by interpolating from available MFI data: two penetrating-medium bulk modulus measurements of H-ZSM-5 (Si/Al = 11.4, K = 27.5 GPa and Si/Al = 20.4, K = 27.4 GPa) [7,8] were used with the experimentally determined bulk modulus of CO_2-loaded silicalite-1 (K = 26 ± 4 GPa) [9]. The interpolation was performed using the aluminium fraction $f_{Al} = 1/(Si/Al +1)$, a physically meaningful compositional coordinate that maps silicalite-1 to $f_{Al} = 0$ and decreases smoothly with increasing Si/Al ratio. A linear model, $K = a \cdot f_{Al} + b$, was fit by ordinary least squares to the three data points. Evaluating the model at f_{Al} corresponding to Si/Al = 33 yields

K = 26.7 GPa. Uncertainty was propagated from the ± 4 GPa experimental bounds of the silicalite-1 measurement by refitting the model using K = 22 and 30 GPa as limiting values. This procedure gives K = 24.7 and 28.7 GPa, defining an uncertainty envelope of ± 2.0 GPa around the nominal estimate. We therefore adopt K = 26.7 ± 2.0 GPa for the strain-energy analysis. Table S1 summarises the input parameters and fitted coefficients.

Table S1. Literature values of Si/Al ratio, aluminium fraction $f_{Al} = 1/(Si/Al + 1)$, and bulk modulus K used to interpolate the bulk modulus of the present H-ZSM-5 crystals (Si/Al = 33). The interpolation employs a linear model $K = a \cdot f_{Al} + b$ fitted to two H-ZSM-5 measurements and one silicalite-1 reference under penetrating-medium conditions.

	**sample**	**Si/Al**	**$f_{Al} = 1/(Si/Al + 1)$**	**K (GPa)**
**$K = a \cdot f_{Al} + b$**<br><br>**a (slope)**<br>**b (intercept)**	H-ZSM-5[1]	11.4	0.081	27.5
	H-ZSM-5[2]	20.4	0.047	27.4
	Silicalite-1[3]	∞	0	26.0 ± 4.0
**interpolation result**	**Present H-ZSM-5**	**33**	**0.029**	**26.7 ± 2.0**

References

[1] Mochizuki, H. *et al.* Facile control of crystallite size of ZSM-5 catalyst for cracking of hexane. *Microporous and Mesoporous Materials* **145**, 165–171 (2011).

[2] Atlan, C. et al. clatlan/cdiutils: v0.1.3. Zenodo. Preprint at https://doi.org/https://doi.org/10.5281/zenodo.7656854 (2023).

[3] Luke, D. R. Relaxed averaged alternating reflections for diffraction imaging. *Inverse Probl* **21**, (2005).

[4] Fienup, J. R. Reconstruction of an object from the modulus of its Fourier transform. *Opt Lett* **3**, 27 (1978).

[5] Gerchberg, R. W. & Saxton, W. O. A practical algorithm for the determination of phase from image and diffraction plane pictures. *Optik (Stuttg)* **35**, 237–246 (1972).

[6] Favre-Nicolin, V., Leake, S. & Chushkin, Y. Free log-likelihood as an unbiased metric for coherent diffraction imaging. *Sci Rep* **10**, 1–8 (2020).

[7] Vezzalini, G., Arletti, R. & Quartieri, S. High-pressure-induced structural changes, amorphization and molecule penetration in MFI microporous materials: A review. *Acta Crystallogr B Struct Sci Cryst Eng Mater* **70**, 444–451 (2014).

[8] Quartieri, S., Montagna, G., Arletti, R. & Vezzalini, G. Elastic behavior of MFI-type zeolites: Compressibility of H-ZSM-5 in penetrating and non-penetrating media. *J Solid State Chem* **184**, 1505–1516 (2011).

[9] Marqueno, T. *et al.* An Ultrahigh CO2-Loaded Silicalite-1 Zeolite: Structural Stability and Physical Properties at High Pressures and Temperatures. *Inorg Chem* **57**, 6447–6455 (2018).
